# A Long-Term Assessment of Nitrogen Removal Performance and Microecosystem Evolution in Bioretention Columns Modified with Sponge Iron

**DOI:** 10.3390/toxics12100727

**Published:** 2024-10-09

**Authors:** Zizeng Lin, Qinghuan Shi, Qiumei He

**Affiliations:** 1 College of Civil Engineering, Nanjing Forestry University, Nanjing 210037, China; 18205197117@163.com; 2 State Key Laboratory of Eco-Hydraulic in Northwest Arid Region of China, Xi’an University of Technology, Xi’an 710048, China; heqiumei27@163.com

**Keywords:** bioretention, sponge iron, long-term operation, nitrogen removal, microecosystem

## Abstract

The nitrogen removal performance of bioretention urgently needs to be improved, and sponge iron has great potential to address this challenge. This study reported the results of a long-term investigation on bioretention columns improved by sponge iron, examining the durability of sponge iron from nitrogen removal performance, sponge iron properties, and the evolution of biological elements. The results showed that after 9 months of continuous operation, the removal rates of ammonia nitrogen (NH_4_^+^-N), nitrate nitrogen (NO_3_^−^-N), and total nitrogen (TN) in the bioretention columns with an appropriate proportion of sponge iron could reach 80% (some even over 90%). However, the long-term stress of sponge iron exposure, combined with the cumulative effect of pollutants, might lead to the excessive accumulation of reactive oxygen species (ROS) in plants, thereby posing risks of diminished chlorophyll content and enzyme activity. Simultaneously, the extended exposure could also have detrimental effects on microbial diversity and the abundance of dominant bacteria such as *Proteobacteria* and *Sphingorhabdus*. Therefore, it is necessary to select plant species and functional genes that demonstrate high adaptability to iron-induced stress.

## 1. Introduction

As a typical green stormwater infrastructure, bioretention has been widely used due to its multifaceted roles in peak flow reduction, runoff quality enhancement, groundwater recharge, and aesthetic environmental enhancement [1]. Bioretention demonstrates good performance in rainwater purification through the joint action of fillers, vegetation, and microorganisms. It effectively eliminates various pollutants, including total suspended solids, heavy metals, total phosphorus, etc. [2,3]. However, nitrogen pollutants, owing to their complex transformation pathways and removal mechanisms, have unstable removal effects in bioretention [4,5] and are greatly affected by factors such as design parameters, operating conditions, inflow concentration, and rainfall intensity [6].

The removal of nitrogen pollutants by bioretention can be achieved by multiple processes including plant uptake, filler adsorption, and microbial involvement in nitrification, denitrification, and anaerobic ammonia oxidation [7]. Under the specific configuration conditions of bioretention, the main constraint on the denitrification effect is the absence of a carbon source and anaerobic environment [8,9]. Zero-valent iron (ZVI) has gained much attention in recent years and is considered to effectively solve the problem of limited denitrification efficiency caused by insufficient carbon sources. This is mainly because ZVI can intervene in both the chemical and biological denitrification processes. On the one hand, ZVI’s superior reductive properties allow it to serve as an electron donor in chemical denitrification reactions (as shown in chemical reactions formulas (1)–(4)), leading to the reduction of NO_3_^−^-N to N_2_, NH_4_^+^-N, or NO_2_^−^-N [10]. Furthermore, the reaction byproduct, Fe^2+^, can participate in Fe(II)-mediated autotrophic denitrification [11]. On the other hand, zero-valent iron can not only utilize H_2_ produced by electrochemical corrosion as an electron donor for specific denitrifying bacteria, thereby promoting the conversion of NO_3_^−^-N to N_2_ [12], but also enrich the nitrogen-removing functional bacterial community within the system [13,14].

(1) $$5{Fe}^{0}+2{NO}_{3}^{-}+6H_{2}O\to 5{Fe}^{2+}+N_{2}↑+12{OH}^{-}$$

(2) $$4{Fe}^{0}+{NO}_{3}^{-}+7H_{2}O\to 4{Fe}^{2+}+{NH}_{4}^{+}+10{OH}^{-}$$

(3) $${Fe}^{0}+{NO}_{3}^{-}+H_{2}O\to {Fe}^{2+}+{NO}_{2}^{-}+2{OH}^{-}$$

(4) $$3{Fe}^{0}+{NO}_{2}^{-}+8H^{+}\to 3{Fe}^{2+}+2H_{2}O+{NH}_{4}^{+}$$

Sponge iron is a new type of zero-valent iron material with high ZVI content. Compared to other zero-valent iron materials, its loose and porous structure endows it with a larger specific surface area, higher specific surface energy, and stronger adsorption capabilities. The nitrogen removal performance of sponge iron has been confirmed in various studies [15,16,17], but its application in bioretention is still lacking. In addition, bioretention is a microecosystem that requires long-term operation, and the metallic properties of sponge iron pose certain risks of stress and toxicity to plants and microorganisms, particularly during prolonged exposure [18,19]. Therefore, it is essential for the practical application of sponge iron in bioretention to verify the effect of long-term nitrogen removal, along with its impact on biological elements, but this is still unknown.

Based on the above knowledge gaps, this study conducted a 9-month indoor continuous operation of sponge iron-improved bioretention columns. The primary objectives were to investigate (i) the sustained nitrogen removal performance, (ii) changes in the physical and chemical properties of sponge iron after use, (iii) changes in the physiological properties of plants, and (iv) transformations in microbial community characteristics. It is hoped that this study can provisde a valuable reference for the practical utilization of sponge iron in bioretention.

## 2. Materials and Methods

### 2.1. Experimental Device

**Figure 1:**
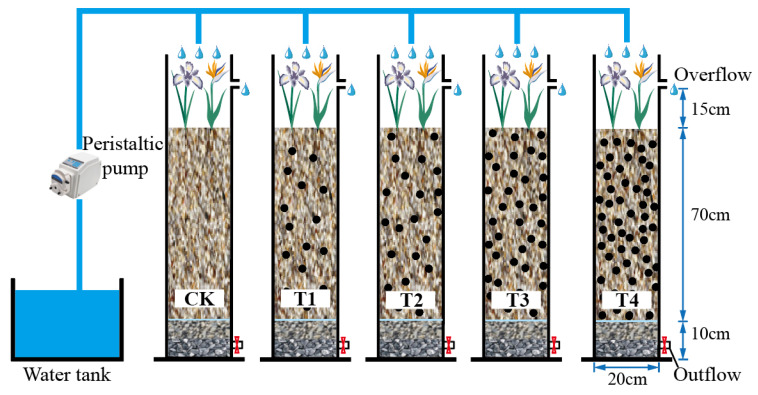
Bioretention device.

As shown in Figure 1, five 20 cm × 100 cm plexiglass columns were used in the experiment, with overflow ports located 5 cm from the top and outlets positioned 2 cm from the bottom. Each column was constructed with a 5 cm gravel drainage layer, a 5 cm coarse sand transition layer, and a 70 cm mixed media layer. The main matrix, composed of a mixture of fine sand, peat soil, and vermiculite in a mass ratio of 20:1:1.05, was combined with sponge iron at various mass ratios (0:1, 1:8, 1:6, 1:4, and 1:2) to fill the five columns labeled CK, T1, T2, T3, and T4, respectively. Five columns were inoculated with microorganisms using activated sludge from a sewage treatment plant in Nanjing. Based on local plant priority, drought resistance, and waterlogging tolerance, *Canna* and *Iris* were planted on the surface of the media, and a 12 h light and 12 h shade alternation was maintained with plant lamps to ensure the normal photosynthesis of plants.

### 2.2. Experimental Scheme

The simulated rainfall experiment was carried out from 1 April 2022 to 1 January 2023, and lasted about 9 months. Tap water dissolved with NH_4_Cl, NaNO_3_, KH_2_PO_4_, and C_6_H_12_O_6_ was used to prepare artificial rainwater with concentrations of NH_4_^+^-N, NO_3_^−^-N, TN, total phosphorus (TP), and chemical oxygen demand (COD) at 3.5, 7.5, 11, 1.2, and 150 mg/L, respectively. The artificial rainwater was used to simulate rainfall on a monthly basis to investigate the denitrification effect of the bioretention columns. In addition, five supplementary stages of experiments were designed to assess the denitrification efficiency of the bioretention columns. In Phases I to III, the operations were carried out with varying initial NO_3_^−^-N levels (8, 16, 32 mg/L, respectively) and a COD/N of 5. Phase IV involved the use of organic carbon-free artificial rainwater to investigate the NO_3_^−^-N removal performance. In Phase V, a high COD/N ratio of 10 was used to evaluate the heterotrophic denitrification capacity of the columns.

According to the rainstorm intensity formula (Formula (5)) [6], this study simulated rainfall events with a 2-year return period and a 120-minute duration. The discharge ratio (catchment area/bioretention surface area) was 10:1, and there was a 3-day dry period prior to each rainfall event.

(5) $$q=\frac{10716.700\left(1+0.837lgP\right)}{{\left(t+32.900\right)}^{1.011}}$$

where *q* is the raisnfall intensity (L/(s·ha)), *P* is return period of rainfall (year), and *t* is rainfall duration (min).

### 2.3. Sample Analysis

During each rainfall event, water samples were collected at 0.5 h, 1 h, 2 h, 3 h, 4 h, 5 h, and 6 h from the time of drainage. The concentrations of NH_4_^+^-N, NO_3_^−^-N, NO_2_^−^-N, and TN were analyzed using sodium reagent spectrophotometry (HJ 535-2009), N-(1-naphthalene)-ethylenediamine colorimetry, UV spectrophotometry (HJ/T 346-2007), and alkaline potassium persulfate digestion UV spectrophotometry (HJ 636-2012), respectively.

On the 63rd and 275th days of operation, the leaves of *Canna* and *Iris* in each column, as well as media samples, were collected for physiological and microbiological analysis. Measurements of H_2_O_2_, malondialdehyde (MDA), chlorophyll, and iron content in the leaf cells were taken using the iron xylenol orange method, thiobarbituric acid method, spectrophotometry, and ortho-phenanthroline method, respectively. The enzyme-linked immunosorbent assay kit (MLBIO, Shanghai, China) and microplate reader (Multiskan FC, Thermo Fisher, Waltham, MA, USA) were used to analyze the activity of superoxide dismutase (SOD), catalase (CAT), ascorbate peroxidase (APX), and peroxidase (POD).

The microbial community structures in the media samples were analyzed using high-throughput sequencing technology. The microbial DNA was extracted using the HiPure Soil DNA extraction kit (Magen, Guangzhou, China). The 16s rRNA V3–V4 region was amplified by the universal primer sequence 341F/806R (CCTACGGGNGGCWGCAG/GGACTACHVGGGTATCTAAT), and then the messages were analyzed.

Considering the overall benefits of a bioretention column with a 1:6 sponge iron addition, both in terms of nitrogen removal performance and engineering cost, sponge iron particles were retrieved from T2 for a durability assessment after 9 months of operation. The chemical composition and surface morphology of the particles were analyzed using an X-ray diffractometer (Ultima IV, Rigaku, Akishima, Japan) and a scanning electron microscope (Quanta 200, FEI, Hillsboro, OR, USA) equipped with an Energy Dispersive Spectrometer (AXIS UltraDLD, Shimadzu, Kyoto, Japan).

## 3. Results

### 3.1. Nitrogen Removal Performance

#### 3.1.1. Removal of Ammonia Nitrogen, Nitrate Nitrogen, and Total Nitrogen

**Figure 2:**
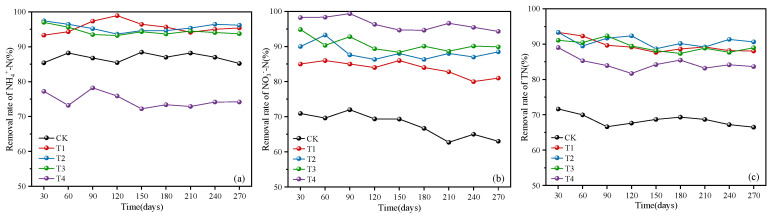
Long-term removal effect of (**a**) NH_4_^+^-N, (**b**) NO_3_^−^-N, and (**c**) TN in the five bioretention columns.

As shown in Figure 2, after 270 days of operation, it was observed that the removal rates of NH_4_^+^-N, NO_3_^−^-N, and TN in five columns slightly decreased by 0.21–3.27%, 1.51–7.93%, and 2.11–5.37%. However, the NO_3_^−^-N and TN in sponge iron-enhanced columns could still be maintained at over 80%, and the NH_4_^+^-N removal rates of certain columns could still exceed 90%, indicating a relatively stable nitrogen removal effect.

Throughout the entire operation period, CK was able to reduce more than 85% of NH_4_^+^-N in the influent. T1, T2, and T3 increased this value by 5.09–13.48%, while T4 reduced it by 8.57–16.24%. The removal rates of NO_3_^−^-N in CK, T1, T2, T3, and T4 were 62.70–71.99%, 82.79–86.01%, 86.32–93.26%, 88.36–94.84%, and 94.31–99.36%, respectively. The removal efficiency showed an increasing trend with the rise in sponge iron dosage, which was consistent with the previous research results [20]. CK achieved a TN removal rate of 66.49–71.64%, while T1, T2, and T3 significantly elevated this value by 18.03–25.71%, followed by T4. The above results indicate that an optimal dosage of sponge iron (such as ratios of 1:8, 1:6, 1:4) could promote the nitrogen removal effect of bioretention, while excessive sponge iron might have adverse effects.

The filler adsorption and microbial transformation processes play pivotal roles in nitrogen removal within bioretention [21]. The vermiculite in the media used in this study has been proven to be an excellent adsorbent for NH_4_^+^-N and NO_3_^−^-N due to its well-developed layered structure and active centers [3,22]. The addition of sponge iron fostered the creation of an anaerobic–anoxic–aerobic microenvironment, which was conducive to the proliferation of microorganisms such as ammonia-oxidizing bacteria, nitrifying bacteria, and denitrifying bacteria in the columns [23]. This, in turn, promoted the conversion of NH_4_^+^-N and NO_3_^−^-N into other substances, resulting in a further reduction of effluent concentrations.

#### 3.1.2. Denitrification Efficiency

**Figure 3:**
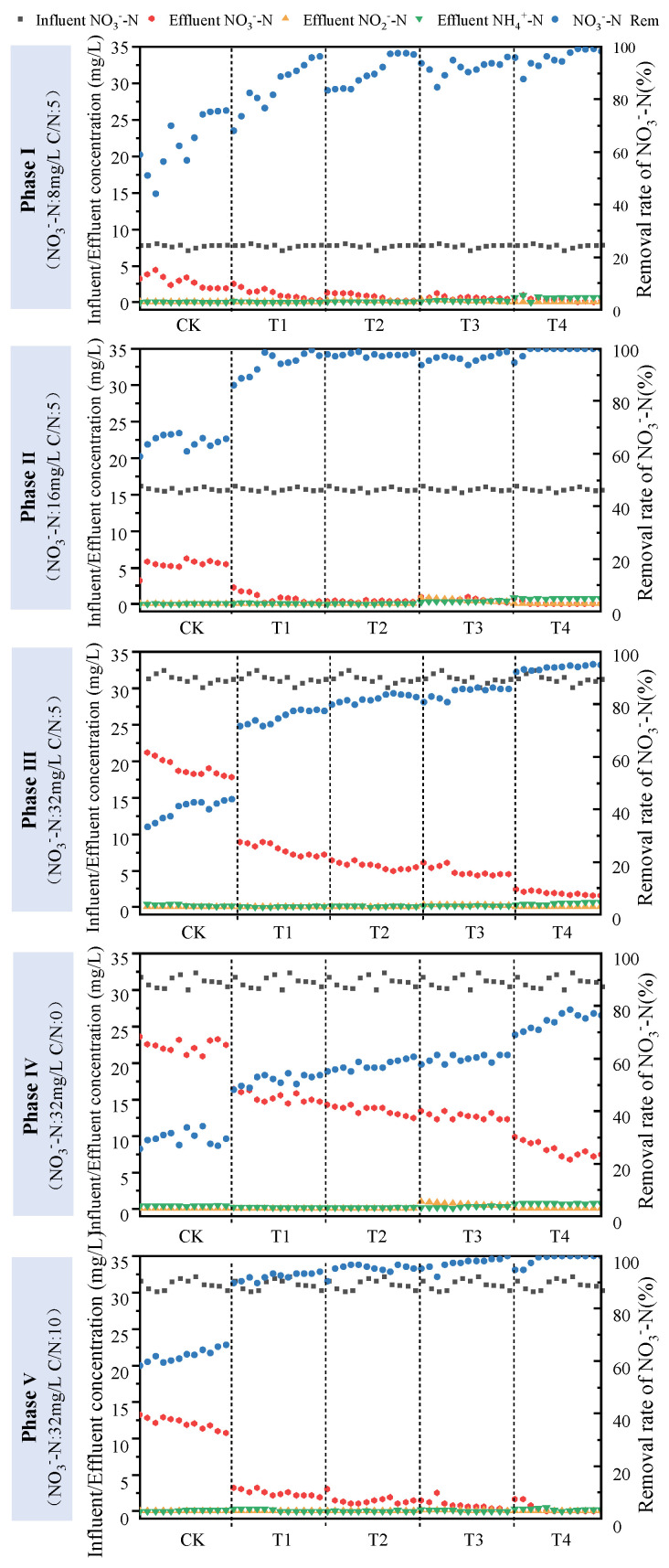
Removal and conversion of NO_3_^−^-N under different influent concentrations of NO_3_^−^-N and initial COD/N ratios in five bioretention columns.

In Figure 3, Phases I–III investigated the NO_3_^−^-N conversion characteristics under varying influent concentrations of NO_3_^−^-N. At an influent concentration of 8 mg/L, the stable removal rates of the five columns were 75.82%, 96.46%, 97.02%, 96.18%, and 98.41%, respectively. When the influent concentration increased to 16mg/L, the stable removal rates became 65.75%, 97.42%, 98.39%, 98.80%, and 100%, respectively. The columns modified by sponge iron showed a slight increase in removal efficiency. However, as the initial concentration further increased to 32 mg/L, the NO_3_^−^-N removal rates significantly decreased by 5.04–21.76%. Nevertheless, the advantage of sponge iron remained evident, with the NO_3_^−^-N removal rates consistently exceeding 78%. T3 and T4 contained relatively high amounts of NH_4_^+^-N (0.26–0.54 mg/L and 0.73–0.81 mg/L, respectively), along with a minor presence of NO_2_^−^-N in Phases I–II. This was because high doses of sponge iron created an increase in electron donors, promoting the reduction of NO_3_^−^ to N_2_ or NH_4_^+^ [24,25], as well as a small amount of NO_3_^−^ to NO_2_^−^ conversion. In Phase III, the rising NO_3_^−^ concentration stimulated these reactions, resulting in a notable increase in NH_4_^+^-N and NO_2_^−^-N in the effluent across all five columns.

Phases IV and V assessed the heterotrophic denitrification ability of sponge iron bioretention whether there was an external carbon source. Notably, the stable NO_3_^−^-N removal rates were lower in the absence of external organic matter (ranging from 29.42% to 76.60%) as compared to the inclusion of supplemental organic matter (ranging from 65.41% to 100%). The lack of a carbon source limited heterotrophic denitrification, with Fe^0^ serving as the primary electron donor to drive chemical denitrification. Consequently, a higher dosage of sponge iron offered a compensatory measure for carbon source deficiency, resulting in a lower NO_3_^−^-N effluent concentration. Additionally, it is worth noting the potential presence of autotrophic denitrification in Phase IV, according to Hu et al [26]. As the COD/N ratio increased from 0 to 10, it was observed that NH_4_^+^-N and NO_3_^−^-N gradually decreased to below 0.3 and 0.1mg/L, respectively, because heterotrophic denitrification played a dominant role and chemical denitrification weakened when sufficient carbon sources were available. However, sponge iron still exerted remarkable denitrification ability, leading to an increase of over 27% in NO_3_^−^-N removal rates, and T4 even completely removed NO_3_^−^-N from the influent, which was similar to the findings of Si et al. [9].

### 3.2. Changes in Surface Morphology and Element Composition of Sponge Iron

**Figure 4:**
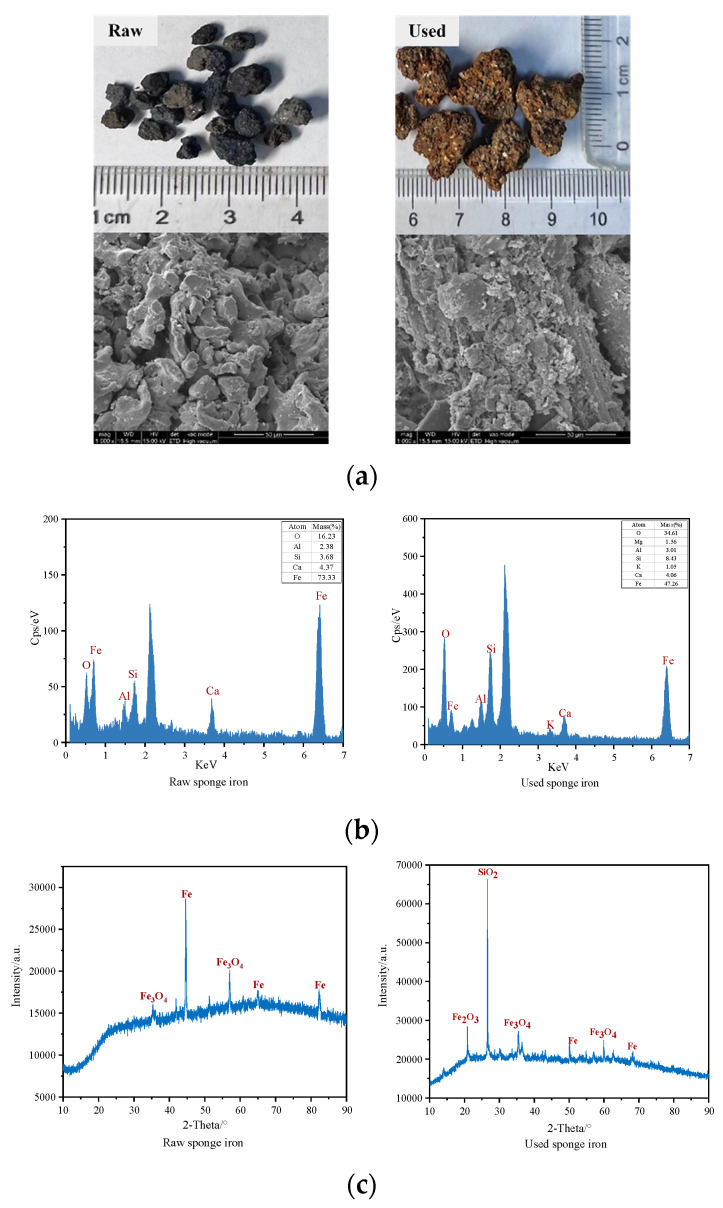
Surface characterization of raw and used sponge iron particles: (**a**) appearance and SEM images, (**b**) EDS scanning images, (**c**) XRD spectra.

Figure 4 showed the appearance, SEM images, EDS scanning images, and XRD spectra of sponge iron particles in the bioretention before and after use. Initially, the sponge iron exhibited a black-gray appearance with a diameter of 3–6 cm, featuring a coral-like surface structure with irregular pores. After 9 months of use, a visible shift occurred as the sponge iron transformed into a yellow-brown hue and underwent a noticeable increase in volume, forming large aggregates on the surface (Figure 4a). In Figure 4b, the EDS scanning results revealed a reduction of approximately 26% in the Fe mass fraction on the surface of sponge iron particles after use, accompanied by an approximate 20% increase in the O mass fraction. This phenomenon was also observed in research on the application of sponge iron in constructed wetlands [9], which might be due to the fact that the water purification process involving sponge iron was accompanied not only by the dissolution and loss of iron ions, but also by the generation of iron oxides adhering to the particle surfaces. According to the XRD analysis (Figure 4c), the surface of sponge iron particles was primarily covered by Fe_3_O_4_ and Fe before use. After 9 months, new Fe_2_O_3_ and SiO_2_ were detected, with a significant enhancement in the Fe_3_O_4_ signal peaks and a slight reduction in the Fe signal peaks. This phenomenon can be attributed to the oxidation of Fe to Fe_2_O_3_ or Fe_3_O_4_ during exposure to water and oxygen, as well as during the denitrification processes. The SiO_2_ signal peak might come from vermiculite adhering to the iron particles.

### 3.3. Changes in Plants’ Physiological Characteristics

#### 3.3.1. MDA, H_2_O_2,_ and Chlorophyll

**Figure 5:**
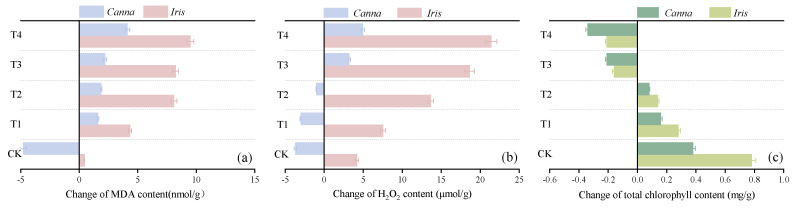
Changes in the content of (**a**) MDA, (**b**) H_2_O_2,_ and (**c**) chlorophyll in the plants of the five bioretention columns.

When subjected to stress, plants produce a large amount of superoxide free radicals which trigger oxidative stress reactions, consequently leading to the accumulation of H_2_O_2_ and MDA [27]. Once the cells are exposed to a peroxidized environment, plant photosynthesis significantly decreases, chlorophyll production is reduced, and plant growth is severely affected, or even stopped [28,29]. Therefore, MDA, H_2_O_2_, and chlorophyll contents are indicative of the impact of stress on plant growth. The changes in these three indicators for both *Iris* and *Canna*, following a 9-month exposure to sponge iron-induced stress, are illustrated in Figure 5.

As depicted in Figure 5a,b, the changes in MDA and H_2_O_2_ in *Canna* were much smaller than those in *Iris*, especially in CK, T1, and T2, where a negative increase in MDA or H_2_O_2_ content was even observed in *Canna*. This suggested that *Canna* could gradually adapt to a limited dosage of sponge iron through its own regulatory mechanism during long-term growth. Furthermore, a higher dosage of sponge iron intensified the peroxidation environment within the plants, triggering the oxidative stress response of plants [30], which could explain the trend of MDA and H_2_O_2_ changes in both plants increasing with the dosage of sponge iron. Chlorophyll content was the core measurement of the plants’ photosynthetic activity, reflecting the photosynthetic capacity and growth status of plants [31]. Over the span of 9 months, the chlorophyll content of *Canna* and *Iris* showed an upward trajectory in CK, T1, and T2, with the highest increments of 0.38 mg/g and 0.78 mg/g, respectively, in CK. However, the chlorophyll content of both plants in T3 and T4 decreased consistently, indicating that excessive sponge iron exposure might seriously damage the photosynthetic performance and inhibit plant growth under long-term stress, and Rios et al.’s research also confirmed this point [32].

#### 3.3.2. Changes in Enzyme Activity

**Figure 6:**
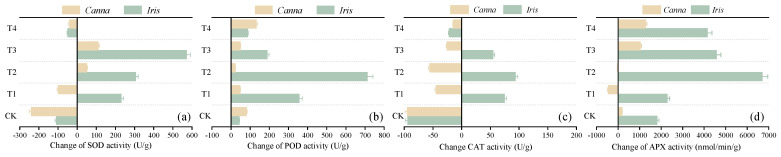
Changes in the activity of (**a**) SOD, (**b**) POD, (**c**) CAT, and (**d**) APX enzymes in the plants of the five bioretention columns.

External stressors could induce antioxidant responses in plants, thereby enhancing the activity of key antioxidant enzymes, such as SOD, POD, CAT, and APX. This activation of the enzymatic defense system serves to prevent the excessive accumulation of ROS, which could otherwise harm the biofilm or other macromolecular substances of plants [33]. SOD can dismutate O_2_^−^ into O_2_ and H_2_O_2_ to inhibit the generation of O_2_^−^, while enzymes such as CAT, POD, and APX are responsible for clearing H_2_O_2_ [34,35]. Figure 6 illustrates the variations in the activity of four antioxidant enzymes in *Canna* and *Iris* exposed to sponge iron-induced stress over a 9-month period.

The SOD activity of *Iris* exhibited significant increases in T1, T2, and T3, with increments of 230.61, 306.21, and 572.0 U/g, respectively, while *Canna* experienced increments of 50.21 and 110.64 U/g in T2 and T3, respectively. Conversely, two plants in other systems displayed a decline in SOD activity. In fact, the antioxidant enzyme system of plants operates within a certain range, and excessive stress intensity and duration could lead to a reduction in enzyme activity [36], which might be associated with damage to cell structure and the degradation of enzyme-related proteins caused by excessive accumulation of ROS [37]. Similarly, the CAT and APX activities of the plants in certain columns exposed to sponge iron for 9 months also demonstrated a decrease, especially in *Canna*, where CAT activity consistently decreased in all five systems (ranging from 14.73 to 94.61 U/g). In contrast, the POD enzyme activity of both plant species was enhanced due to its higher affinity for H_2_O_2_ and its ability to participate in the formation of lignin, strengthening the resilience of plants to iron-induced stress through stiffened cell walls [38]. Similar conclusions were obtained in the study of *Vallisneria natans (Lour.) Hara* under ammonia-induced stress [39].

#### 3.3.3. Accumulation of Iron in Plants

**Figure 7:**
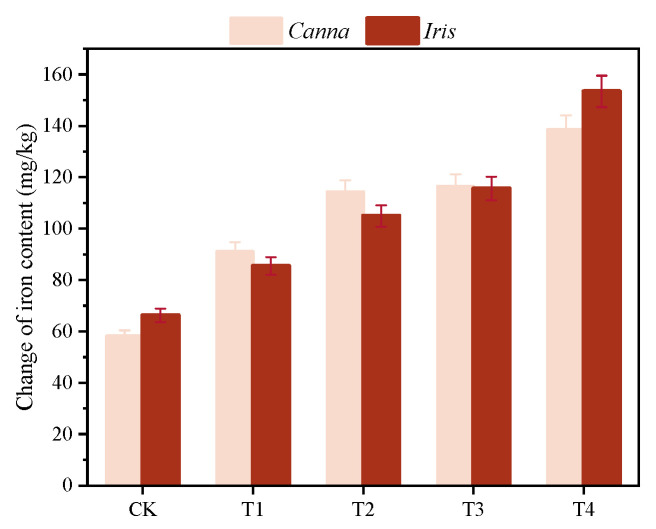
Accumulated iron content of the plant leaves in the five bioretention columns.

Iron plays a vital role in the physiological processes within plants, such as chlorophyll synthesis, respiration, and redox reactions. When plants undergo iron deficiency, it results in a decline in chlorophyll content and subsequent leaf yellowing [40]. Excessive iron accumulation can lead to iron toxicity in plants. This toxicity is attributed to the generation of ROS triggered by intracellular free Fe^2+^, which in turn damages cellular structure and function [41]. This study analyzed the cumulative iron content in the leaves of *Canna* and *Iris* after a 9-month exposure to sponge iron, as illustrated in Figure 7.

The iron content in the leaves of both *Canna* and *Iris* demonstrated a clear trend with T4 > T3 > T2 > T1 > CK, highlighting an obvious dependency on the dosage of sponge iron added. *Canna* and *Iris*, as monocotyledonous gramineae plants, mainly rely on the mechanism of chelating Fe^3+^ for absorption to acquire iron elements from soil [42]. Consequently, an augmented dosage of sponge iron resulted in an increased presence of Fe^2+^/Fe^3+^ within the media, which was subsequently absorbed by the plants. This finding aligns with that of Pan et al. [43], where an analogous increase in the total accumulation of Cd in *Canna* tissue was observed in response to a higher Cd supply.

The iron content needed to sustain normal plant growth is 100–300 mg/kg, but the total iron content in general soils seldom exceeds 10^−15^ mg/kg, and even does not surpass 10^−17^ mg/kg, especially in alkaline soil [44]. The total iron content in the leaves of *Canna* and *Iris* in CK and T1 was less than 100 mg/kg, while in T2, T3, and T4, it fell within the range required for plant growth. Even the highest dosage of sponge iron did not cause iron toxicity to the plants.

### 3.4. Changes in Functional Microorganisms for Nitrogen Removal

#### 3.4.1. Microbial Diversity

**Figure 8:**
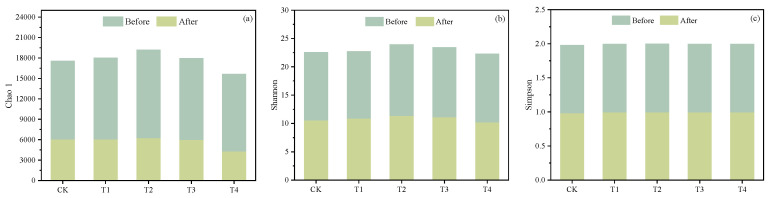
Microbial diversity indices in the five bioretention columns before and after 9 months of operation: (**a**) Chao 1, (**b**) Shannon, (**c**) Simpson.

The Goods coverage index value was above 0.99 in each column, substantiating the authenticity and representativeness of the sequencing outcomes [8]. As depicted in Figure 8, the Chao 1, Shannon, and Simpson indices were used as indicators for measuring microbial species diversity. After 9 months of operation, a decrease in microbial diversity was observed in each bioretention column, with a significant decline in the Chao 1 index, which might be linked to the accumulation of pollutants [45].

#### 3.4.2. Microbial Community Structure at the Phylum and Genus Levels

Table 1 displays the relative abundance of diverse bacterial species within the five bioretention columns, categorized at the phylum and genus levels. At the phylum level, the Proteobacteria, which play an important role in the removal of nitrogen, phosphorus, and many organic compounds [46], showed the highest relative abundance level in all five columns (over 26%). After 9 months, except for the negligible changes in the relative abundance of *Proteobacteria* in T1 and T2 (−2.31% and 1.13%, respectively), other columns significantly decreased by 5.95–16.24%. The fluctuations in the abundance of *Bacteroidetes* and *Patescibacteria* were significant, with the highest variations in CK and T1 (close to ±10%), but the variations manifested opposing trends. After 9 months, there was a notable increase of 13.47% in *Bacteroidetes* in T4, while *Patescibacteria* remained relatively stable. Some *Planctomycetes* have been demonstrated to be involved in the process of reducing NO_3_^−^ to convert it into N_2_ or other nitrogen-containing compounds under hypoxic conditions [47], and the relative abundance of *Planctomycetes* in the five columns did not show significant changes, with 5.97–7.63% before the experiment and 8.53–9.86% afterward. Similarly, there were no obvious changes in *Acidobacteria*, *Actinobacteria*, *Chloroflexi*, and *Firmicutes*, except for *Actinobacteria* and *Firmicutes* in T4, which exhibited changes in the relative abundance of −6.97% and 10.37%, respectively, over 9 months. In fact, the synergistic or competitive effects among microbial populations, the accumulation of pollutants, and various environmental factors might lead to an increase or decrease in the relative abundance of microbial bacteria [48].

There were significant differences in microorganisms at the genus level among the five columns. For example, *Sphingorhabdus* dominated at 7.09% in CK before the experiment and was also detected in other columns; however, it was conspicuously absent in all five columns after the experiment. *Sphingorhabdus* has a good survival ability under low nutrient conditions [49], but the long-term accumulation of nitrogen, phosphorus, and organic matter in this study might have inhibited its growth. After a duration of 9 months, *Terrimonas* and *Acidibacter* still persisted in a relatively dominant position within the CK, T1, T2, and T3. Similarly, the dominance of *Hydrogenophaga* and *Nitrospira* in T4 remained largely unaffected following long-term operation. In addition, *Thiobacillus*, *Thauera*, *Pseudomonas*, and *Dechloromonas*—common bacterial genera of heterotrophic denitrification [50]—had relatively low abundance levels in most columns, even less than 0.1% in some cases. This indicates the limited effectiveness of heterotrophic denitrification in the bioretention columns of this study, while showing the enhancement of chemical denitrification with the introduction of sponge iron.

## 4. Conclusions

This study investigated the long-term performance of sponge iron used in bioretention ponds and its impact on habitat factors. Bioretention with an appropriate dosage of sponge iron consistently maintained a denitrification efficiency of 80% or even 90% during 9 months of operation. The stress of long-term exposure to sponge iron could cause decreases in the chlorophyll content and enzyme activity in plants. However, the accumulated iron content in the leaves of the plants investigated in this study remained within acceptable limits, with no significant detrimental effects observed. The accumulation of pollutants could affect microbial diversity and abundance. Therefore, in practical engineering, it is essential to select plant species and functional microbial communities that are well adapted to iron-induced stress.

## Figures and Tables

**Table 1:** Proportion (%) of dominant bacteria at the phylum and genus levels in the five bioretention columns before and after the experiment.

	Proportion (%)	CK		T1		T2		T3		T4	
Item		Before	After	Before	After	Before	After	Before	After	Before	After
Phylum level	Proteobacteria	36.32	30.37	27.99	25.68	31.49	32.62	37.69	28.94	42.52	26.28
	Bacteroidetes	18.23	9.30	13.81	22.31	12.74	11.89	10.73	15.09	6.43	19.91
	Patescibacteria	7.69	16.42	20.76	11.73	10.93	6.27	10.99	9.17	3.68	4.12
	Planctomycetes	6.77	9.49	6.21	8.56	5.97	8.53	6.35	9.27	7.63	9.86
	Acidobacteria	6.37	8.88	6.43	5.53	8.02	8.40	6.58	7.30	5.03	3.94
	Actinobacteria	1.72	2.86	2.44	2.79	2.78	4.85	5.16	3.01	13.82	6.85
	Chloroflexi	3.72	6.83	3.64	6.27	4.52	8.65	3.91	9.05	4.13	4.27
	Firmicutes	0.58	1.61	0.55	1.76	0.94	2.24	0.62	2.73	1.24	11.61
Genus level	Sphingorhabdus	7.09	-	0.50	-	0.29	-	0.82	-	0.74	-
	Terrimonas	2.22	1.75	1.91	0.96	1.39	1.79	1.47	2.23	0.62	0.58
	Acidibacter	1.83	1.46	0.94	1.03	0.93	0.93	0.74	0.86	0.30	0.25
	Nitrospira	0.62	0.45	0.78	0.52	0.90	0.92	0.95	1.87	0.71	1.18
	Thiobacillus	0.01	0.01	0.01	0.02	0.01	0.13	0.01	0.04	0.14	0.13
	Thauera	0.02	0.21	0.13	0.08	0.01	0.32	0.06	0.19	0.09	0.65
	Pseudomonas	0.62	0.78	0.38	0.21	0.28	0.13	0.26	0.09	0.14	0.09
	Dechloromonas	0.10	0.23	0.22	0.17	0.07	0.15	0.21	0.13	0.27	0.02
	Hydrogenophaga	0.22	0.15	0.57	0.61	0.78	0.67	1.02	0.97	0.88	0.91

## Data Availability

The original contributions presented in the study are included in the article; further inquiries can be directed to the corresponding author.
